# Enalapril-Induced Angioedema: Two Case Reports in a Rural Health Facility in Kenya

**DOI:** 10.7759/cureus.2572

**Published:** 2018-05-02

**Authors:** Mitchel Okumu, Francis Ochola, Calvin Bodo, Kevin Apuoyo, Nelson Odhiambo, Albert Ng'ong'a

**Affiliations:** 1 Department of Pharmacy, Jaramogi Oginga Odinga Teaching and Referral Hospital; 2 Department of Pharmacology and Toxicology, Moi University School of Medicine; 3 Department of Clinical Medicine, Nyakach County Hospital; 4 Department of Pharmacy, Nyakach County Hospital

**Keywords:** enalapril, kenya, rural, angioedema, ace inhibitor

## Abstract

Tolerability, a good safety profile, affordability, and a preponderance to afford cardio-renal protection in patients with diabetes make enalapril one of the most commonly prescribed angiotensin-converting enzyme (ACE) inhibitors. However, there is low awareness of enalapril/ACE inhibitor-induced angioedema among medical personnel. This is because the diagnosis presents an ongoing challenge, particularly when the presentation is delayed following long-term therapy with ACE inhibitors. Here, we present two cases: a 58-year-old female and a 55-year-old male who presented to the outpatient department of Nyakach County Hospital, Pap Onditi village, Kenya, with progressive swelling of the face and upper and lower lips and stridor of 11 and 10 hours, respectively, after their usual dose of enalapril. Case 1 resolved following the administration of stat doses of intravenous (IV) hydrocortisone 200 mg and IV chlorpheniramine 20 mg as well as thrice daily peroral doses of chlorpheniramine 8 mg, and tapered peroral doses of prednisolone: 40 mg thrice daily for five days, 20 mg thrice daily for five days, 10 mg thrice daily for five days, and 5 mg thrice daily for five days. Case 2 resolved following the administration of a stat dose of IV dexamethasone, a twice daily peroral dose of cetrizine 10 mg, and tapered peroral doses of prednisolone: 20 mg thrice daily for five days, 10 mg thrice daily for five days, and 5 mg thrice daily for five days.

## Introduction

Enalapril is an angiotensin-converting enzyme (ACE) inhibitor that is used in the treatment of hypertension, renal failure, myocardial infarction, and diabetic nephropathy [1]. However, not only is angioedema a rare side-effect of this class of drugs, but it is also largely under-recognized [2]. Retrospective studies (mainly postmarketing type) estimate the incidence of ACE inhibitor-induced angioedema to be between 0.1% and 0.7%, while prospective clinical trials estimate the incidence to be anywhere between 2.8% and 6.0% [3]. Risk factors of ACE inhibitor-associated angioedema include advanced age, female gender, smoking, organ transplantation, rheumatoid arthritis, history of ACE inhibitor-associated cough, heart failure, atopy, seasonal allergies, and the concurrent use of ACE inhibitors with nonsteroidal anti-inflammatory drugs (NSAIDs), 3-hydroxy-3-methylglutaryl coenzyme A (HMG-COA) reductase inhibitors, and immunosuppressants [4]. Symptoms begin anywhere from one day to 10 years after initiation of ACE inhibitor therapy [4]. In view of the fact that enalapril-induced angioedema is a rare and potentially life-threatening condition, it is important that clinicians make the correct diagnosis of this adverse effect. We report two cases of enalapril-induced angioedema in a rural healthcare setting in Kenya.

## Case presentation

Case 1

**Figure 1:**
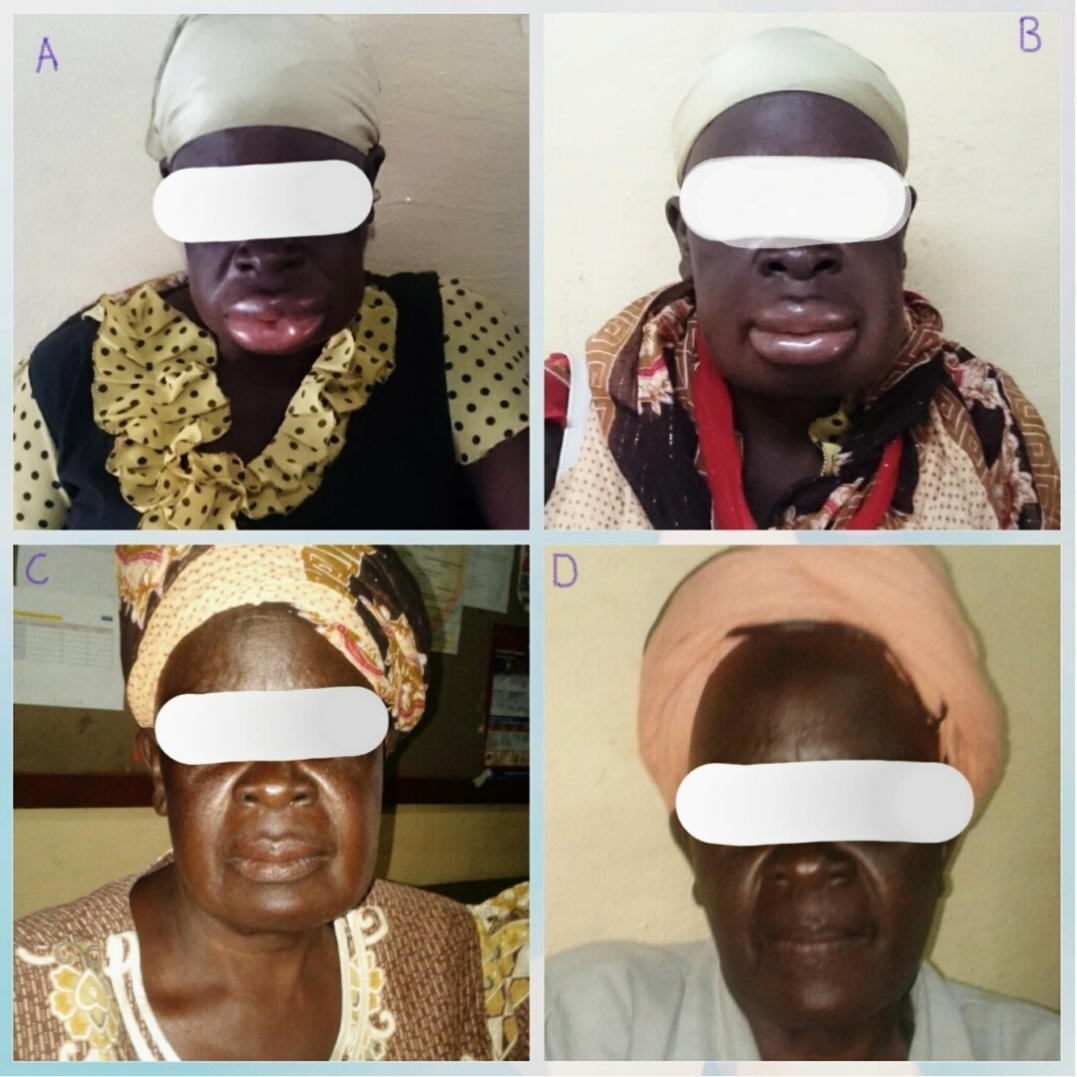
Enalapril-induced angioedema in a female patient A. Angioedema of the face, upper and lower lips at presentation. B. One hour post administration of intravenous hydrocortisone and intravenous chlorpheniramine. C. One week after receiving a tapered peroral dose of prednisolone and a peroral dose of chlorpheniramine. D. Three weeks after receiving a peroral dose of prednisolone and a peroral dose of chlorpheniramine. Permission to use these images was granted by the patient.

A 58-year-old woman with a four-year history of hypertension-diabetes comorbidity presented to the outpatient department of the Nyakach County Hospital with edematous swelling of the face and upper and lower lips of 11-hour duration (Figure 1A).

There was associated dysphagia with stridor and hoarseness of voice. She did not have any pruritus, urticaria, or rashes. The tongue was swollen and was reported as hard in consistency. It was wedged between her teeth which prevented her from closing her mouth. Saliva was pooling and dribbling from her mouth. The patient had no history of smoking, angiotensin-converting enzyme (ACE) inhibitor-induced cough, atopy, or any recent use of aspirin or nonsteroidal anti-inflammatory drugs (NSAIDs). Additionally, there was no prior history of a similar episode. The outpatient card indicated that the patient had tolerated a twice daily peroral dose of metformin 500 mg, a once daily peroral dose of glibenclamide 5 mg, a once daily peroral dose of hydrochlorothiazide 50 mg, and a once daily per oral dose of enalapril 5 mg for four years. Other aspects of her medical history were unremarkable. On examination, she had a pulse rate of 75 beats per minute, respiratory rate of 26 breaths per minute, and blood pressure of 140/72 mmHg. Pulse oximetry was not done. Her systemic examination was unremarkable. Review of her medication profile prompted us to suspect enalapril as the cause of the angioedema. Thus, we made use of the Naranjo probability scale to evaluate the likelihood that the observed effect was enalapril induced. Specific responses on this scale were one, two, one, zero, two, zero, zero, zero, zero, one for a cumulative score of seven, which ranks as probable. Therefore, a preliminary diagnosis of enalapril-induced angioedema was made and the enalapril was immediately discontinued. A 200 mg stat intravenous (IV) dose of hydrocortisone and a 20 mg stat dose of intravenous chlorpheniramine were administered and the patient was observed for one hour. The edema was noted to subside (Figure 1B) and a further three hour period of observation was allowed. The patient was then discharged  on a once daily peroral dose of nifedipine 20 mg, a thrice daily peroral dose of chlorpheniramine 8 mg, and a tapered peroral dose of prednisolone as follows: 40 mg thrice daily for five days, 20 mg thrice daily for five days, 10 mg thrice daily for five days, and 5 mg thrice daily for five days. In addition, she was advised to continue with her usual doses of metformin, glibenclamide, and hydrochlorothiazide and to report to the hospital immediately if the swelling was to re-occur. On follow-up after one week, there was a marked change in facial appearance (Figure 1C), and at three weeks, the patient was symptom-free with a blood pressure (BP) of 142/78 mmHg (Figure 1D).

Case 2

**Figure 2:**
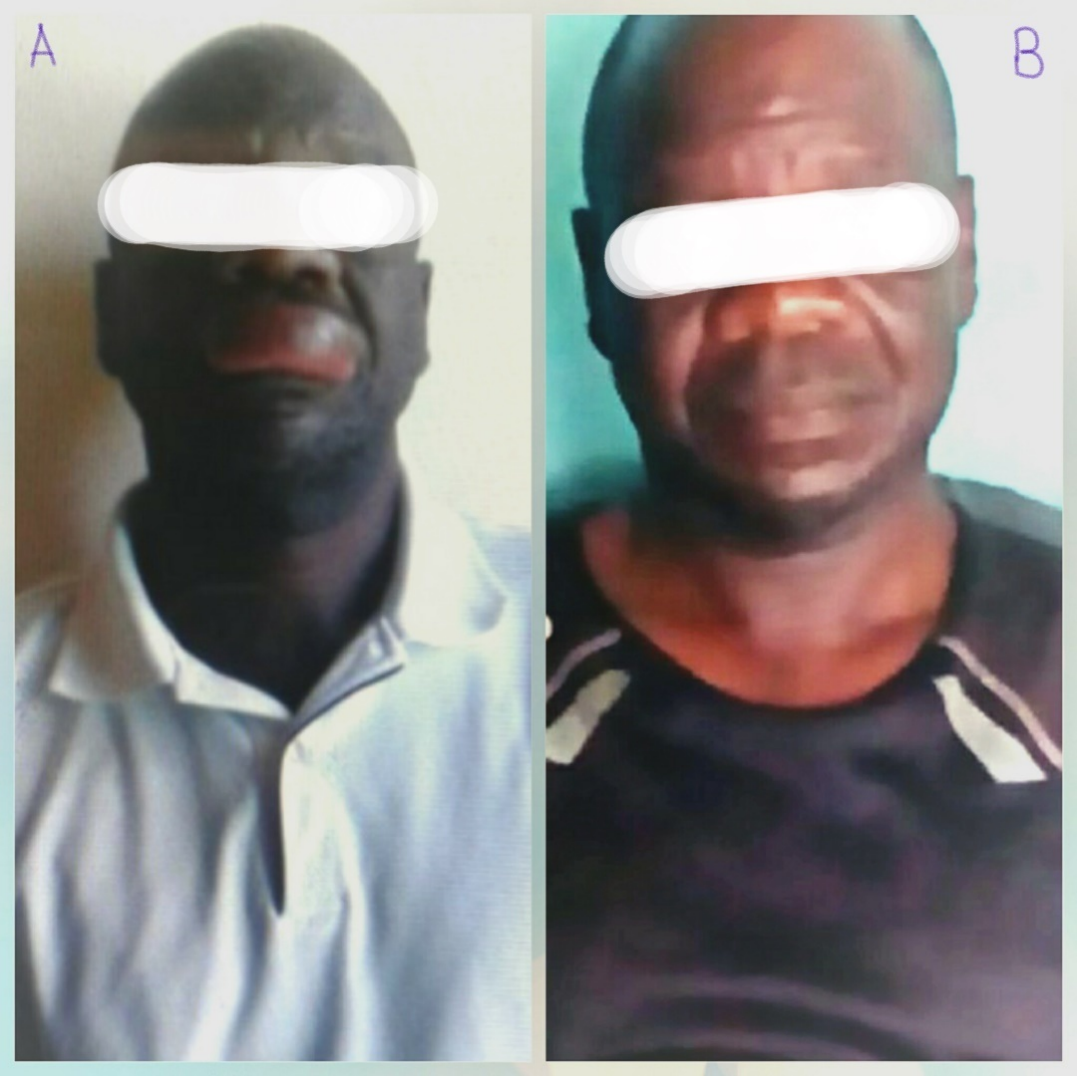
Enalapril-induced angiodema in a male patient A. Angioedematous swelling of the upper lip on presentation. B. Resolution of swelling three weeks after initiation of a peroral dose of prednisolone and a peroral dose of cetirizine. Permission to use these images was granted by the patient.

A 55-year-old male with a six-year history of hypertension presented to the Nyakach County Hospital with swelling of the upper lip of 10-hours duration (Figure 2A). The patient complained of restlessness and insomnia. There was no history of atopy or food or drug allergies. On examination, vitals were normal. Moreover, cardiovascular, respiratory, and abdominal examinations were unremarkable. The outpatient card indicated that he had tolerated nifedipine 20 mg, enalapril 5 mg, and hydrochlorothiazide 25 mg for six years. We suspected enalapril to be responsible for the edema and thus made use of the Naranjo probability scale to evaluate whether the observed edema was enalapril induced. The responses on this scale were one, two, one, zero, two, zero, zero, zero, zero, one for a total score of seven, which ranks as probable. Therefore, a preliminary diagnosis of enalapril-induced angioedema was made, enalapril withheld, and the patient immediately put on 4 mg intravenous dexamethasone injection and monitored for one hour. A gradual decrease in the swelling was observed about two hours after the intravenous dose of dexamethasone. The patient was discharged on a peroral dose of prednisolone tapered as follows: a thrice daily 20 mg dose for five days, a thrice daily 10 mg dose for five days, and a thrice daily 5 mg dose for five days. A twice daily peroral dose of cetirizine 10 mg was also given for 10 days, and the patient was advised to continue taking the nifedipine and hydrochlorothiazide, but to report immediately to the hospital if the swelling re-occured. After one week, the patient was reviewed and it was observed that there was a significant resolution of the swelling. On subsequent follow-up after three weeks, it was observed that the patient was symptom-free (Figure 2B).

## Discussion

We reviewed the patient’s medication profile to evaluate possible drug-related causes of angioedema. The medications were examined for a relationship between the initiation of treatment and the onset of angioedema. The adverse effect profiles of the drugs and cases of similar reactions were also considered. Based on these criteria, enalapril, nifedipine, and hydrochlorothiazide were identified as probable causes of the observed angioedema. However, given the relationship between treatment initiation and the onset of angioedema (four and six years respectively) in our cases, as well as other case reports associating angioedema with ACE inhibitor use in individuals of African descent [5-6], we considered enalapril to be the most probable cause of the observed angioedema. Both patients scored 7/10 on the Naranjo probability scale, further corroborating our suspicion of enalapril as the cause of the observed angioedema. This prompted the immediate withdrawal of the drug in both cases. Treatment with metformin, glibenclamide, and hydrochlorothiazide was continued for the first case as was nifedipine and hydrochlorothiazide in the second case. Owing to the unnecessary risk of airway obstruction we would be posing to these patients, we made a conscientious decision not to re-challenge them with what we presumed was the offending drug. Instead, we promptly initiated corticosteroid (prednisolone, dexamethasone, hydrocortisone) and antihistamine (cetirizine, chlorpheniramine) therapy. 

There has been one documented case of metformin-induced angioedema [7]. However, we ruled this out on the premise that this adverse effect was reported to occur during the first dose of metformin. In our case, the first patient had been on metformin for more than three years. Moreover, the fact that no angioedema was observed in our patients with continued use of metformin after enalapril was withdrawn may be considered further evidence that the angioedema could not have been caused by metformin. Also, ACE inhibitor-induced angioedema has been associated with increased levels of c-reactive protein, an inflammatory marker [7]. On the contrary, metformin has been associated with a decrease in c-reactive protein and soluble intercellular adhesion molecule (si-CAM) [7] and thus may not have been the cause of angioedema in our case.

There have been reports of amlodipine and hydrochlorothiazide-induced angioedema [8-9], but these were also ruled out given the fact that no angioedema was observed following the use of these drugs post enalapril de-challenge.

ACE inhibitor-associated angioedema often involves the lips, tongue, face, and throat. However, swelling of the genitalia, abdomen, viscera, and lower extremities have also been reported [6]. When it involves the bowel wall, patients may present with nausea and vomiting, abdominal pain, diarrhea, or ascites [6]. The ACE inhibitor-induced angioedema may at times be self-limiting, but when the swelling occurs in the head and neck regions, particularly the pharynx and larynx, emergency care and hospitalization may often be required [5].

The ACE inhibitor-induced angioedema is a consequence of decreased degradation of bradykinin as well as other vasoactive ACE derivatives such as substance P. Bradykinin is responsible for increased vascular permeability through its B2 receptor as well as via sensitization of the transient vanilloid receptor I (TRPVI) [10].

Recognition and discontinuation of the offending ACE inhibitor are the mainstays in the primary management of ACE inhibitor-induced angioedema [6]. Other primary therapy measures include management of the airway, and possibly intubation or cricothyroidotomy [6]. The use of antihistamines, corticosteroids, and epinephrine by physicians to manage ACE inhibitor-associated angioedema is common but there is an absence of clinical trial data that shows that the use of these agents is efficacious [6]. Several studies suggest that it is safe to treat such patients with angiotensin receptor blockers (ARBs) [6]. However, since angioedema may recur anywhere between one day and three weeks after discontinuation of an ACE inhibitor, it is recommended that a six-week window is allowed before any ARB is used [6].

The two patients improved significantly with supportive therapy. However, in as much as these cases lacked a complement 1 (C1) inhibitor (C1-INH) function test to exclude the possibility of hereditary angioedema or acquired C1-INH deficiency, and having ruled out all other possible drug-related angioedema, the presenting symptoms of face, lip, and tongue swelling accompanied with the pooling and drooling of saliva are hallmarks of ACE inhibitor-induced angioedema. These cases show that despite the widely known safety of ACE inhibitors, potentially life-threatening side-effects do exist.

## Conclusions

Clinicians and pharmacists in rural and urban settings alike should be aware of the likelihood of occurrence of angioedema in patients on long-term enalapril therapy. The prompt identification of enalapril/ACE inhibitor-induced angioedema and its subsequent management are key in preventing further deterioration of the condition to a life-threatening event. Patients should also be informed of the risk of angioedema as a rare side-effect of using enalapril. 

## References

[1] Adebayo PB, Alebiosu OC (2009). ACE-I induced angioedema: a case report and review of literature. Cases J.

[2] Sarkar P, Nicholson G, Hall G (2006). Brief review: angiotensin converting enzyme inhibitors and angioedema: anesthetic implications. J Can Anesth.

[3] Kostis JB, Packer M, Black HR, Schmieder R, Henry D, Levy E (2004). Omapatrilat and enalapril in patients with hypertension: the Omapatrilat Cardiovascular Treatment vs. Enalapril (OCTAVE) trial. Am J Hyperten.

[4] Sinert R, Levy P, Bernstein JA, Body R, Sivilotti MLA, Moellman J (2016). Randomized trial of icatibant for angiotensin-converting enzyme inhibitor-induced upper airway angioedema. J Allerg Clin Immunol Prac.

[5] Stone C, Brown NJ (2017). Angiotensin-converting enzyme inhibitor and other drug-associated angioedema. Immunol Allerg Clin North Am.

[6] Baram M, Kommuri A, Sellers SA, Cohn JR (2013). ACE inhibitor-induced angioedema. J Allerg Clin Immunol Prac.

[7] de Oliveira JRJM, Otuki MF, Cabrini DA, Brusco I, Oliveira SM, Ferreira J (2016). Involvement of the TRPV1 receptor in plasma extravasation in airways of rats treated with an angiotensin-converting enzyme inhibitor. Pulmon Pharmacol Ther.

[8] Atik D, Büyükcam F, Yılmaz D, Işık B, Demir ÖF (2013). Angioedema after the first dose of metformin. Am J Emerg Med.

[9] Southward J, Irvine E, Rabinovich M (2009). Probable amlodipine-induced angioedema. Ann Pharmacother.

[10] Ruscin JM, Page II RL, Scott J (2006). Hydrochlorothiazide-induced angioedema in a patient allergic to sulfonamide antibiotics: evidence from a case report and a review of the literature. Am J Geriatr Pharmacother.

